# IL-15 deficiency alleviates steroid-induced osteonecrosis of the femoral head by impact osteoclasts via RANKL-RANK-OPG system

**DOI:** 10.1186/s12979-020-00190-0

**Published:** 2020-06-12

**Authors:** Zubin Zhou, Yiwei Lin, Chenhao Pan, Nan Wang, Lihui Zhou, Haojie Shan, Youshui Gao, Xiaowei Yu

**Affiliations:** 1 Department of Orthopaedic Surgery, Shanghai Jiao Tong University Affiliated Sixth People’s Hospital, Shanghai, 200233 China; 2 Department of Emergency, the First Affiliated Hospital of Zhengzhou University, Zhengzhou, 450052 Henan China; 3 Department of Orthopaedic Surgery, Xiangshan First People’s Hospital, Ningbo, 315700 Zhejiang China

**Keywords:** IL-15, Osteonecrosis, RANKL, RANK, OPG

## Abstract

**Background:**

Whether IL-15 is involved in the development of steroid-induced osteonecrosis of the femoral head (ONFH) is investigated.

**Methods:**

C57BL/6 J and l15^−/−^mice were injected with methylprednisolone to induce wide type osteonecrosis (WT ON) and IL-15 deficiency osteonecrosis (IL-15^−/−^ ON). Hematoxylin-Eosin (H&E) staining and micro-computed tomography (micro-CT) scanning was used to detect the microstructure. The differentiation and formation of osteoclasts were determined with colony-forming unit-granulocyte macrophages (CFU-GM), colony-forming unit-macrophage/mononuclear (CFU-M) per tibia, and tartrate-resistant acid phosphatase (TRACP or TRAP) positive cells. Serum interleukin (IL)-15, osteocalcin, bone alkaline phosphatase (BAP), bone Gla protein (BGP), and TRACP were assayed with enzyme-linked immunosorbent assay (ELISA). The receptor activator of nuclear factor-κB (RANK), RANK ligand (RANKL), and osteoprotegerin (OPG) in the femoral heads were detected by Western blot. CD34 staining was performed to detect microvascular density.

**Results:**

IL-15 secretion was increased in the femoral heads and the serum of steroid-induced ONFH mice. IL-15 deficiency may lead to up-regulated vessel remodeling, improved microstructure, and up-regulated serum osteocalcin, BAP, and BGP secretion. Both the expression of RANKL/RANK/OPG and osteoclast differentiation and formation can be down-regulated by IL-15 deficiency.

**Conclusion:**

IL-15 deficiency alleviates steroid-induced ONFH by impact osteoclasts via RANKL-RANK-OPG system.

## Introduction

Osteonecrosis, also called avascular necrosis, is the collapse of bone tissue due to lack of blood supply [1, 2] which commonly affects femur. Although osteonecrosis can happen without any apparent reason, high-dose usage of glucocorticoids (steroid hormones), alcoholism, bone fractures, and joint dislocations are considered to be the risk factors. At present medical practice, the steroid is indispensably utilized to treat various diseases, such as nephrotic syndrome, renal transplantation, acute lymphoblastic leukemia, rheumatoid arthritis, and systemic lupus erythematosus, which will be accompanied with the serious complication of steroid-induced osteonecrosis of the femoral head (ONFH) and hip replacement might be the only option at the terminal stage [3–5].

It is worth noting that steroid-induced ONFH usually affects persons from 30 to 50 years old, and 47.4% of non-traumatic ONFH are directly associated with steroid usage in a Japanese epidemiology study [6, 7]. In consideration of the serious medical consequences and enormous economic costs incurred by ONFH, it is urgent and vital to find an effective option to improve bone repair and inhibit articulus collapse.

IL-15 has been testified to promote the differentiation of osteoclast progenitors into preosteoclasts [8]. And it is recently reported that IL-15 could stimulate osteoblasts apoptosis via NK cells [9, 10]. Since the balance between osteoblasts and osteoclasts may play a vital role in bone formation, whether IL-15 deficiency may inhibit the development of steroid-induced ONFH is investigated.

## Methods & materials

### Mice

C57BL/6NTac/Il15^−/−^ mice obtained from Taconic Farms were backcrossed to C57BL/6 J background (Peking Vital River Laboratory Animal Ltd., Beijing, China) to construct C57BL/6 J/Il15^−/−^(presented as Il15^−/−^) mice for 4 generations. There was no mortality can be attributed to backcross manipulation. All mice were maintained and operated in specified pathogen-free conditions according to the 8th edition of the National Institutes of Health Guide for the Care and Use of Laboratory Animals (2011) [11]. The experiment procedure was approved by the Ethics Committee of Shanghai Jiao Tong University Affiliated Sixth People’s Hospital.

### Groups and treatment

C57BL/6 J mice and Il15^−/−^ mice were injected with methylprednisolone (21 mg/kg) subcutaneously for 4 consecutive weeks to set up steroid-induced ONFH groups as osteonecrosis WT group (WT ON) and osteonecrosis IL15^−/−^ group (IL15^−/−^ ON) as the previous report recommended [12], while C57BL/6 J mice and Il15^−/−^ mice administrated with physiological saline were utilized as sham group and Il15^−/−^ sham group.

### Tissue sample preparation

After 4 weeks, mice from different groups were intraperitoneally anesthetized with ketamine (1.4 mg/mouse) and xylazine (0.12 mg/mouse), and bilateral femora were obtained. Left femoral was pre-fixed in 4% paraformaldehyde (PFA, pH 7.4) for 72 h, which were further decalcified with ethylenediaminetetraacetic acid (EDTA, 10%, pH 7.4) for 4 weeks. Then the tissues were embedded in paraffin, cross-sectioned into 5 mm sections, stained with hematoxylin and eosin (H&E). Medullary hematopoietic cells necrosis, empty lacunae, and or condensed osteocytes nuclei were utilized to define osteonecrosis. Right femoral were used for Western blots and quantitative real-time PCR assays.

### Micro-CT

A micro-computed tomography (Micro-CT) (GE Healthcare Biosciences, Piscataway, USA) was used to detect changes in the excised femoral head sample and bone trabeculae of 8–10 weeks old mice. The scanning parameters were set at 50 kV, 0.5 mm aluminium filter, 500 μA source current, exposure time 700 ms, 9 μm isotopic resolution, 3 projection images per 0.3° rotation step, and a voxel resolution of 20 μm. For data reconstruction, the NRecon software (v1.6.9.8) was used, with Gaussian smoothing, ring artefact correction and 40% beam hardening correction applied. Using Data viewer software (v1.4.4) each dataset was normalized regarding its orientation and saved in trans axial (X-Y) projections, and then exported to CTAn software (v1.13.11.0). To measure bone microarchitecture parameters for each dataset, growth plate plus 0.25 mm was used as structural reference between each sample and then, using CTAn software, the regions of interest (ROI) for 1.2 mm in length were selected and three-dimensional microarchitecture parameters calculated for both the femur and tibia and expressed according with the ASBMR recommendations. Microarchitecture parameters included bone volume/tissue volume (BV/TV), trabecular number (Tb.N), trabecular thickness (Tb.Th), trabecular separation (Tb.Sp), bone mineral content (BMC), and bone mineral density (BMD).

### Microvessels counting

The intensity of CD34 antigen staining in microvessels per field was counted using the method previously reported [13]. As a transmembrane phosphoglycoprotein, CD34 is generally found in the epithelium of blood vessels. CD34 positive endothelial cells or cell clusters which could be distinguished from adjacent clusters were defined as microvessels. The average number of microvessels identified within the 5 random selected fields was summed up (3 slices, 6 mice for each group).

### Colony-forming unit-granulocyte macrophages (CFU-GM) and Colony-forming unit-macrophage/mononuclear (CFU-M) assay

Bone marrow-derived cells (BMDCs) were flushed from tibias with DMEM containing 2% fetal bovine serum (FBS), which were further separated with low-density gradient Ficoll and seeded at a density of 2.5 × 10^4^ cells with methylcellulose combined with M-CSF (30 ng/ml) and RANKL (20 ng/ml). After the cells were cultured for 7 days at 37 °C in 5% CO_2_ incubator, phase-contrast microscopy was utilized to count the number of colonies [14].

### Osteoclast formation assay

For osteoclast differentiation, BMDCs were seeded at a density of 1 × 10^5^ cells/well into 96-well plates and cultured with MEM-α supplemented FBS (10%), M-CSF (30 ng/ml), and RANKL (20 ng/ml) for 2-day. On the 3rd day, MEM-α supplemented with FBS (10%), M-CSF (30 ng/ml), and RANKL (60 ng/ml) was utilized to incubate the BMDCs for 3 more days and tartrate-resistant acid phosphate (TRAP)-positive multinuclear cells were determined as osteoclasts. Cellsense standard software was utilized to determine the ratio of osteoclasts in five randomly selected fields per well (400×).

### Quantitative real-time RT-PCR (qRT-PCR)

Total RNA extracted from 1 mm thick superficial right femoral head tissues (*n* = 6 per group) with TRIzol reagent was reverse transcribed using a High Capacity cDNA Reverse Transcription Kit (Invitrogen, Carlsbad, CA, USA). QRT-PCR performed with SYBR Green (Roche, Mannheim, Germany) was used to detect IL-15 and β-actin. The setting of the reaction was as follows: 95 °C for 10 min, 95 °C for 15 s (40 cycles), and 60 °C for 1 min. Relative IL-15 expression was quantified using the comparative ΔCT method and normalized to β-actin. Primer sequences were listed: β-actin, forward primer 5′-CTAAGGCCAACCGTGAAAAG-3′, reverse primer 5′- TACATGGCTGGGGTGTTGA -3′; IL-15, 5′- GGCAGCTTGCAGGTCCTCC-3′, reverse primer 5′- CGTCCAACTCTGCAACTGG-3′.

### Western blotting

The 1 mm thick superficial right femoral head tissues (*n* = 3 per group) were lysed and separated by 10% SDS-PAGE. After 5% nonfat dry milk blocking, the transferred PVDF membrane was incubated with the primary antibodies for vascular endothelial growth factor (VEGF), receptor activator of nuclear factor κB (RANK), RANK ligand (RANKL), bone morphogenetic protein (BMP7), osteoprotegerin (OPG), runt-related transcription factor 2 (RUNX2), and glyceraldehyde-3-Phosphate Dehydrogenase (GAPDH) (Abcam, Cambridge, US) at 1:1000 dilution and 4 °C overnight. The membranes were then incubated with the peroxidase-conjugated secondary antibody (Thermo Fisher Scientific, Inc., Rockford, IL, USA) (1:1000 dilution, 1 h, room temperature) and developed with an ECL system (GE Healthcare Life Sciences, Chalfont, UK). The relative intensity was calculated by correcting for GAPDH from the same sample.

### Enzyme-linked immunosorbent assay (ELISA)

The serum IL-15, osteocalcin, bone alkaline phosphatase (BAP), osteocalcin (BGP), TRAP, IL-1β, TNF-α, IFN-γ and IL-6 were measured with ELISA kits (Invitrogen, CA, USA). All standards and samples were assayed with a SpectraMax M5 microplate reader (Molecular Devices) at a wavelength of 450 nm.

### Statistical analysis

Data were expressed as the mean ± the standard deviation (SD) and analyzed by t-test or one-way ANOVA analysis followed by a Tukey’s post hoc test. *P* < 0.05 was considered to be statistically significant.

## Results

### IL-15 contributes to the development of steroid-induced ONFH

**Fig. 1:**
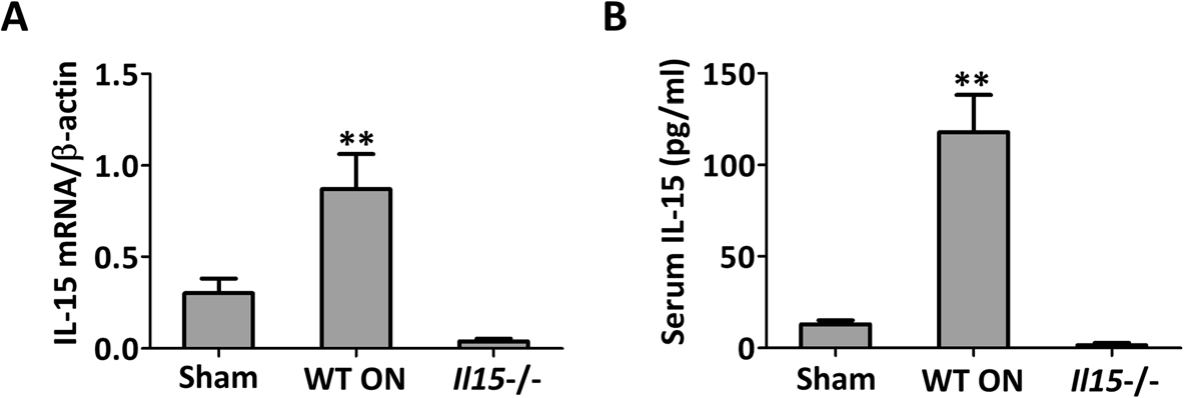
IL-15 levels were increased in the femoral heads and serum of mice with steroid-induced ONFH. **a** IL-15 expression in the femoral heads was assayed with quantitative real-time RT-PCR. **b** IL-15 levels in the serum were detected by ELISA. Sham = operated control group, ON = osteonecrosis group, *Il15*−/− = osteonecrosis IL-15 deficiency group. The data were expressed as means ± SD (*n* = 6). **p < 0.01 versus the ON group

**Fig. 2:**
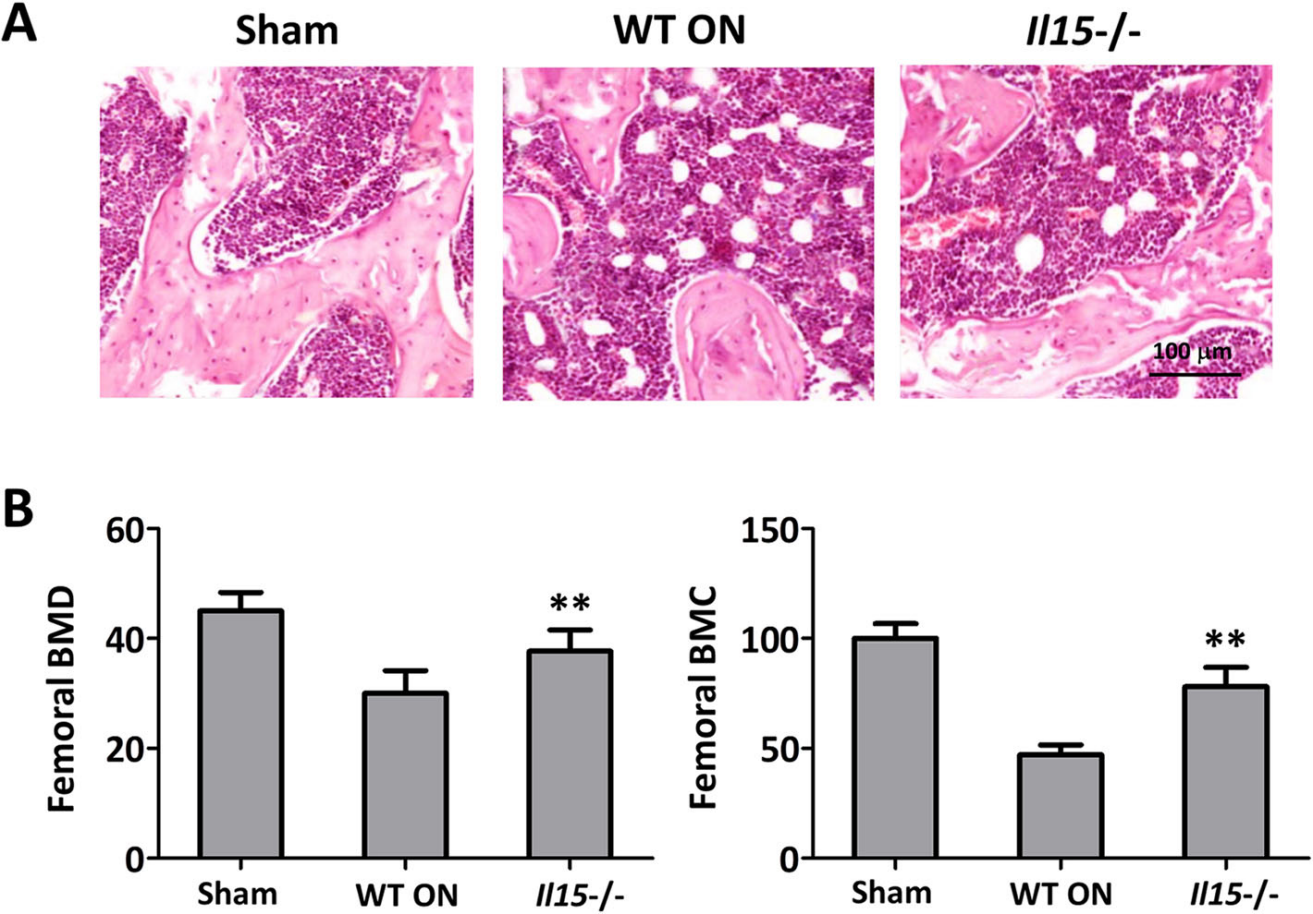
IL-15 deficiency attenuated osteonecrosis in mice with steroid-induced ONFH. **a** The observation of femoral head osteonecrosis by Hematoxylin-Eosin staining. **b** BMD and BMC were improved for IL-15 deficiency. Sham = operated control group, WT ON = osteonecrosis WT group, *Il15*−/− = osteonecrosis IL-15 deficiency group. The data were expressed as means ± SD (*n* = 6). **p < 0.01 versus the WT ON group

After steroid-induced ONFH model was established, IL-15 level was detected. And it was found that both the expression of IL-15 in the femoral heads (Fig. 1a) and the secretion in the serum (Fig. 1b) were increased when compared with the sham group. While the trace amount IL-15 expression in the femoral heads (Fig. 1a) and the secretion in the serum (Fig. 1b) also confirmed the IL15 knockout. The histopathological observation was performed to verify the establishment of steroid-induced ONFH and the involvement of IL-15. It was demonstrated that WT ON group showed characteristic osteonecrosis feature with empty lacunae, marrow cell necrosis, and occupation of adipocytes. While IL-15^−/−^ group showed fewer empty lacunae and adipocytes when compared with WT ON group (Fig. 2a). In addition, femoral BMD and BMC were partly restored in IL-15^−/−^ group when compared with WT ON group (Fig. 2b) (*p* < 0.01).

### IL-15 deficiency attenuates osteonecrosis both in the microstructure and vessel remodeling

**Fig. 3:**
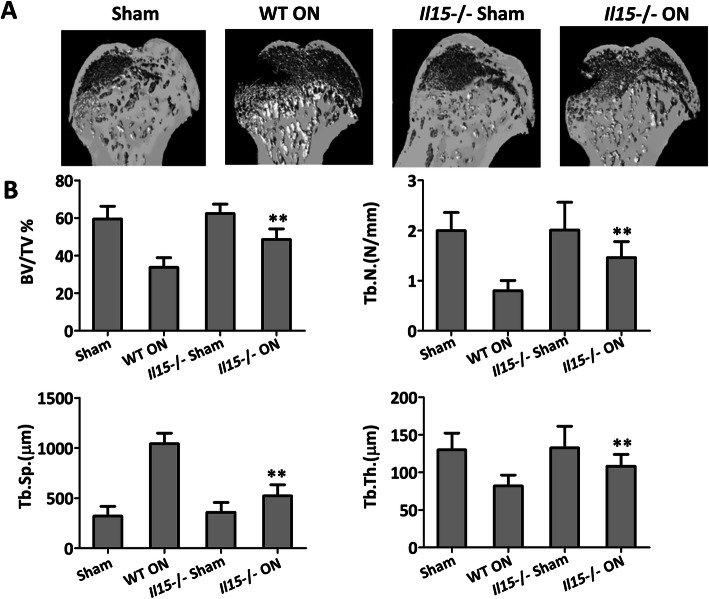
IL-15 deficiency attenuated the microstructure of the femoral head in mice with steroid-induced ONFH. **a** three-dimensional (3D) pictures of femoral head osteonecrosis. **b** Statistical analysis was performed on BV/TV, Tb. N, Tb.sp., and Tb.Th. The data were expressed as means ± SD (*n* = 6). **p < 0.01 versus the WT ON group

It was noted that the mortality of IL15^−/−^ mice with or without methylprednisolone treatment was 11.1 and 10.5%, respectively. Three-dimensional (3D) pictures of the femoral head were reconstructed (Fig. 3a), which further confirmed the H&E observation (Fig. 2a) that IL-15 deficiency can lead to attenuated microstructure osteonecrosis and increased BMD. Such improvements were also indicated by BV/TV, Tb. N, and Tb. Th, which were significantly increased in IL-15^−/−^ group when compared with WT ON group (*p* < 0.01). While, when compared with WT ON group, Tb. Sp was decreased significantly in IL-15^−/−^ group (*p* < 0.01) (Fig. 3b). It was worth noting that there was no significant microstructure difference between sham group and IL-15^−/−^ sham group (Fig. 3a and b), which indicates that IL15 knockout performance did not alter the baseline level of bone mass.

**Fig. 4:**
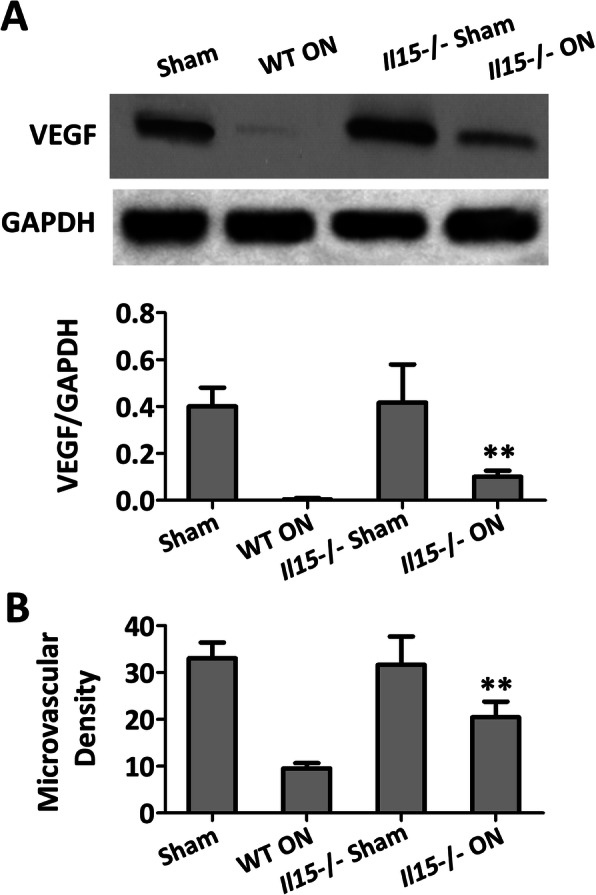
IL-15 deficiency attenuated vessel remodeling of the femoral head in mice with steroid-induced ONFH. **a** VEGF protein level in the femoral head. **b** Microvascular density. The data were expressed as means ± SD (*n* = 6). **p < 0.01 versus the WT ON group

As to microvascular formation, there was no significant difference between the sham group and IL-15^−/−^ sham group (Fig. 4a and b). While consistent with the improvement of the trabecular microstructure, it showed that VEGF was down-regulated significantly in the WT ON group compared with the sham group, and IL-15 deficiency could partly restore the expression of VEGF (Fig. 4a). The intensity of CD34 staining also indicated that IL-15 deficiency could increase the microvascular density (Fig. 4b). All of these results suggested that IL-15 deficiency could prevent bone mass loss and up-regulate microvascular remodeling.

### IL-15 deficiency inhibits RANKL-induced osteoclast differentiation and activates the bone formation

**Fig. 5:**
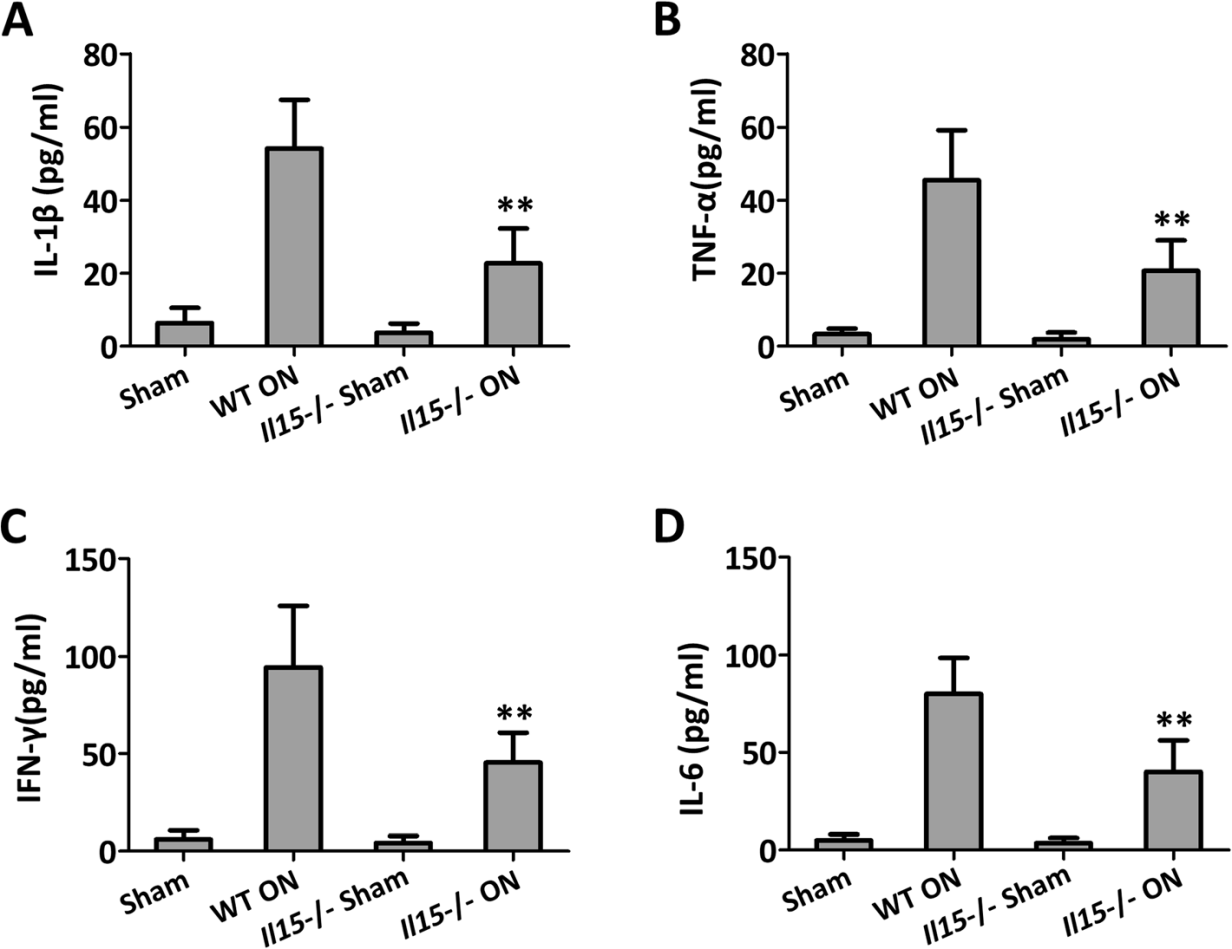
IL-15 deficiency attenuated proinflammatory cytokines in the serum of mice with steroid-induced ONFH. **a** IL-1β; **b** TNF-α, **c** IFN-γ, and **d** IL-6. The data were expressed as means ± SD (*n* = 6). **p < 0.01 versus the WT ON group

The immunological phenotype of IL15 deficiency mice was indicated by the detection of serum cytokine. It clearly showed that there was no significant difference of IL-1β (Fig. 5a), TNF-α (Fig. 5b), IFN-γ (Fig. 5c), and IL-6 (Fig. 5d) between IL-15^−/−^ sham group and the sham group. While as expected, IL15 deficiency showed decreased levels of IL-1β (Fig. 5a), TNF-α (Fig. 5b), IFN-γ (Fig. 5c), and IL-6 (Fig. 5d) when compared with WT ON group.

**Fig. 6:**
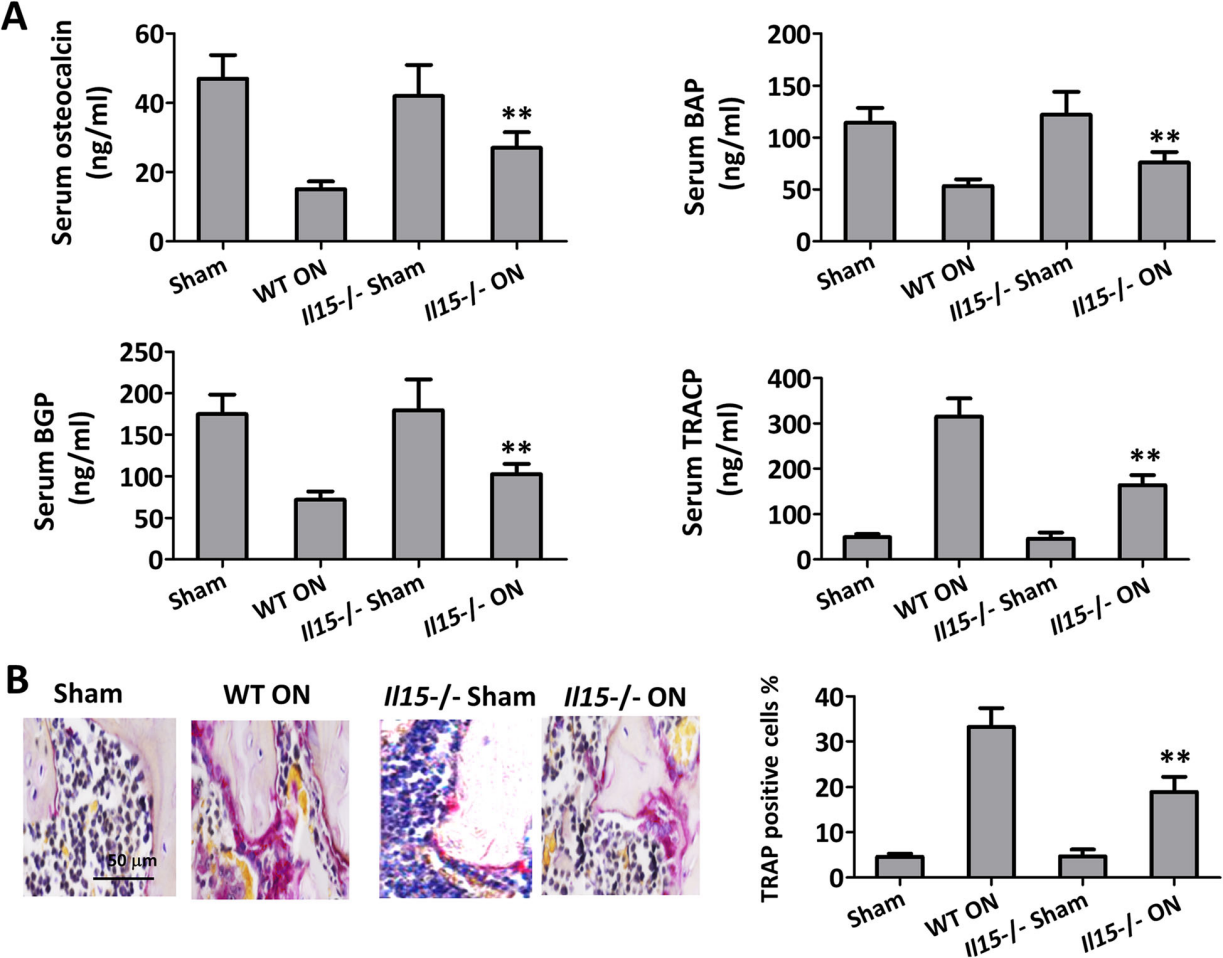
IL-15 deficiency inhibited osteoclast differentiation in mice with steroid-induced ONFH. **a** Serum osteocalcin, BAP, BGP, and TRACP were measured with ELISA. **b** TRAP-positive cells in the femoral head were calculated with immunohistochemical analysis. The data were expressed as means ± SD (*n* = 6). **p < 0.01 versus the WT ON group

Serum osteocalcin, BAP, and BGP can be used as evaluation markers of osteoblast activity, increased significantly in IL-15^−/−^ ON group than WT ON group. Whereas, serum TRACP expressed and secreted by osteoclasts was notably decreased in IL-15^−/−^ ON group compared with WT ON group (Fig. 6a, *p* < 0.01). Osteoclasts, which were defined as TRAP-positive multinucleated cells within the bone destruction area, were significantly decreased in ON treated IL-15 deficiency mice compared with WT ON mice (Fig. 6b, *p* < 0.01).

**Fig. 7:**
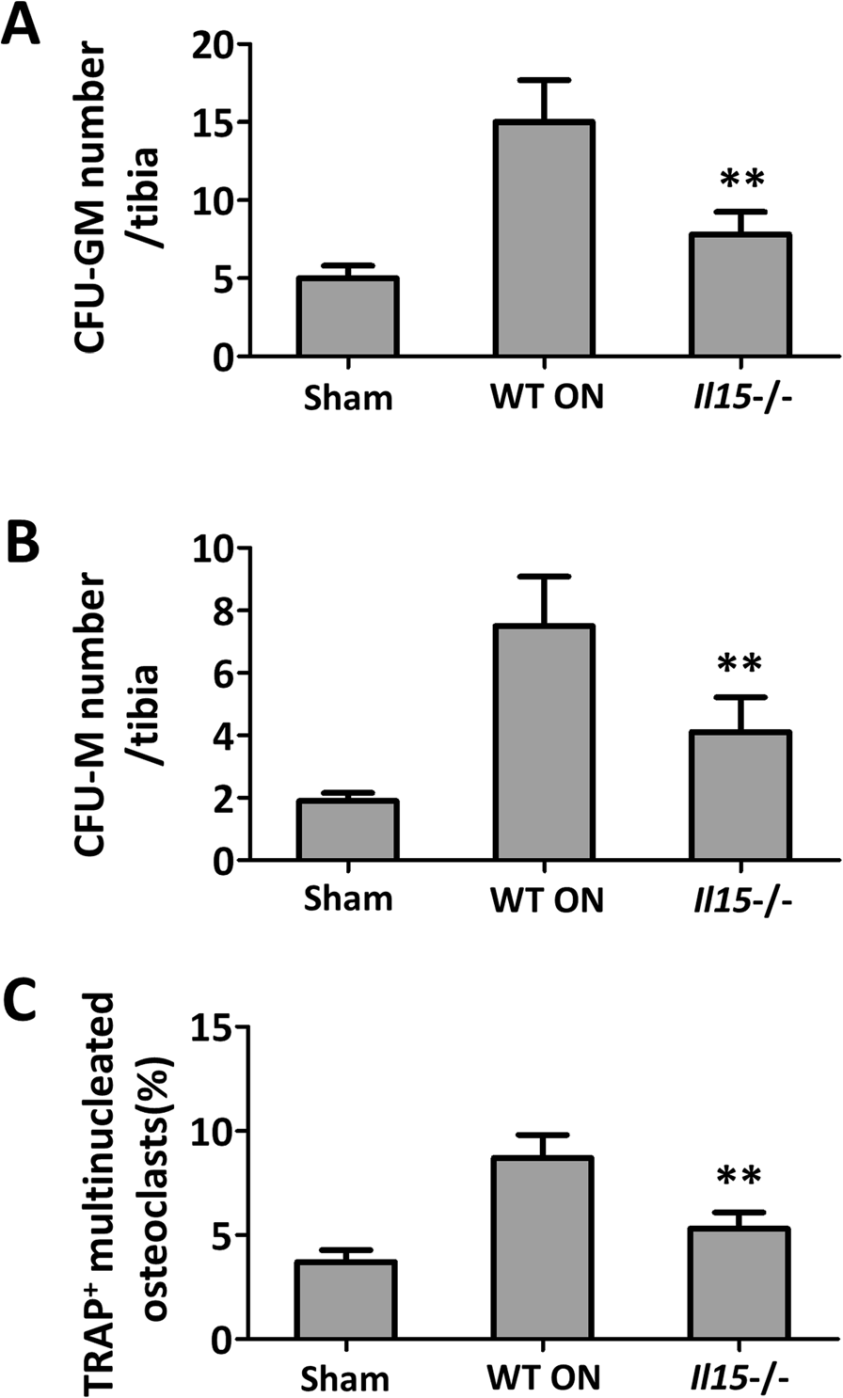
IL-15 deficiency decreased the amount of CFU-GM/CFU-M and inhibited osteoclasts formation. The numbers of CFU-GM (**a**) and CFU-M (**b**) differentiated from bone marrow-derived cells were significantly decreased by IL-15 deficiency. **c** Osteoclast formation increased in the osteonecrosis group, and IL-15 deficiency significantly attenuated the osteoclasts maturation. The data were expressed as means ± SD (*n* = 6). **p < 0.01 versus the WT ON group

When bone marrow-derived cells were incubated with RANKL and M-CSF, IL-15 deficiency reduced the numbers of CFU-GM/CFU-M (Fig. 7a and b) and TRAP-positive osteoclasts (Fig. 7c), which indicated the reduced differentiation and formation of osteoclasts.

### IL-15 deficiency regulates the RANKL/RANK/OPG signaling system

**Fig. 8:**
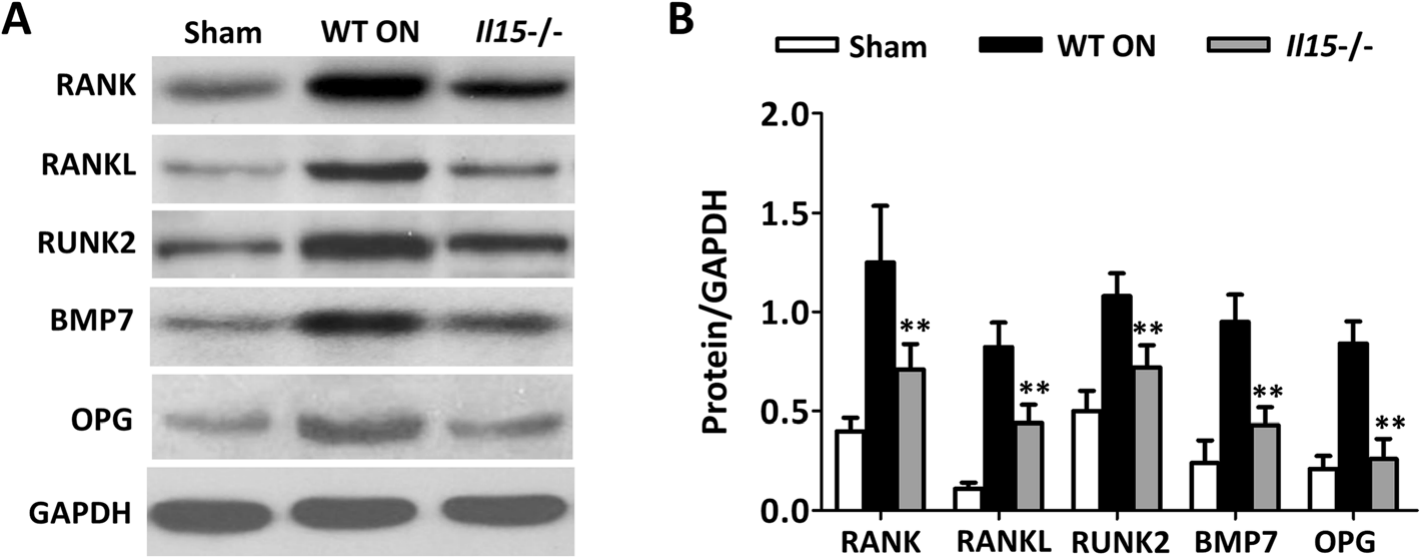
IL-15 deficiency regulates the RANKL/RANK/OPG signaling pathway in mice with steroid-induced ONFH. **a** RANK, RANKL, and OPG levels in the femoral heads were detected at protein levels by western blot. **b** shows the ratio of RANK, RANKL, RUNK2, BMP7 and OPG/GAPDH. The data are expressed as means ± SD(*n* = 3). ** *p* < 0.01 versus the WT ON group

RANKL expressed by osteoblasts can trigger osteoclast maturation and bone resorption by binding with RANK on osteoclasts in the presence of M-CSF, while such interaction can be inhibited by OPG expressed by osteoblasts, which is testified as a decoy receptor for RANKL. RANK, RANKL, RUNX2, BMP7, and OPG levels in WT ON group were prominently higher than the sham control group. In addition, IL-15 deficiency could significantly down-regulate such increase (Fig. 8a and b).

## Discussion

In the current study, IL-15 deficiency could attenuate steroid-induced ONFH by improving the trabecular bone microstructure and enhancing femoral head neovascularization. It is further testified that IL-15 deficiency could inhibit bone marrow-derived RANKL-induced osteoclast differentiation and formation, and regulate RANKL/RANK/OPG signaling to promote the formation of bone in steroid-induced ONFH mice.

Steroid-induced ONFH can lead to the femoral head collapse and subsequent hip joint destroys, which will significantly affect the patients’ activities [15–17]. For steroids could target on multi-system or organs, it is relatively difficult to systemically interpret the mechanism involved in steroid-induced ONFH. It is generally indicated that both the impairment of bone microstructure maintenance by osteoblasts and the promotion of osteoclastic resorption by osteoclasts might be one of the most common biological processes involved in steroid-induced ONFH [18, 19]. The decreased expression of BAP (markers of osteoblast) in the serum and increased TRAP-positive cells in the femoral head indicate that IL-15 deficiency may prohibit osteoclast genesis and promote osteoblast genesis in steroid-induced ONFH mice.

Accumulating investigations have indicated that osteoblasts and osteoclasts can interact through RANKL/RANK/OPG system to mediate bone modelling and remodeling [20, 21], and the relative expression and the balance between RANKL and OPG in osteoblasts can determine the mass and strength of bone, which means the upregulated OPG/RANKL ratio could prevent osteoclastogenesis [22–25]. Although the detailed mechanism is unknown, the up-regulated OPG, RANK, and RANKL in the necrotic femoral head can be reversed by IL-15 deficiency. Whether the upregulated OPG/RANKL ratio or other mechanism involved needs further investigation.

The impaired blood supply and compromise of bone vasculature in the femoral head has been indicated in the progressive development of steroid-induced ONFH, which can lead to the joint destruction within a few months to 2 years. EGF expression and microvascular density in this research are detected to quantify new blood vessel formation. It is worth noting that the RANKL/RANK/OPG system, responsible for ossification and bone mineralization, also contributes to vasculature ossification and vascular compromise [26]. Thus, consistent with the improvement of the trabecular bone microstructure, IL-15 deficiency may also provide a conducive blood supply environment for bone reconstruction. All of these indicate that IL15 may mediate the vital signal pathway in the development of ONFH.

As a widely expressed pro-inflammatory cytokine, IL-15 can participate in NK cells mediated osteoclasts activation and turnover [10, 27]. It is also implicated as a pro-osteoclastogenic cytokine, for IL-15 deficiency can impair osteoclast activity and inhibit trabecular bone loss in ovariectomized mice [28]. Single nucleotide polymorphisms (SNPs) of IL-15 is correlated with cortical bone volume as well as mineral density in humans [29, 30]. Our investigation not only clarifies the inhibitive effects of IL-15 deficiency on RANKL-induced osteoclast formation [31], but also indicates that IL15 inhibition can be utilized as a potential target for osteonecrosis in steroid-induced ONFH patients. But perhaps more significantly, in consideration of the contribution of IL15 in bone resorption and inflammation, IL15 inhibition will also apply to *Staphylococcus aureus*-induced arthritis [32], which can cause rapid joint destruction and disabling joint damage despite antibiotics utilization. All of these indicate the necessity of further detailed mechanism research about IL15 in osteonecrosis.

## Conclusion

IL-15 deficiency can protect steroid-induced ONFH by regulating the RANKL/RANK/OPG signaling. And the inhibition of IL-15 with neutralizing antibody or small-molecule inhibitor can be considered as a potential treatment option.

## Data Availability

The datasets used and/or analyzed during the current study are available from the corresponding author on reasonable request.
